# Nutrient removal from Chinese coastal waters by large-scale seaweed aquaculture

**DOI:** 10.1038/srep46613

**Published:** 2017-04-21

**Authors:** Xi Xiao, Susana Agusti, Fang Lin, Ke Li, Yaoru Pan, Yan Yu, Yuhan Zheng, Jiaping Wu, Carlos M. Duarte

**Affiliations:** 1 Zhejiang University, Ocean College, 1 Zheda Road, Zhoushan, Zhejiang 316000, China; 2 King Abdullah University of Science and Technology (KAUST), Red Sea Research Center (RSRC), Thuwal, 23955-6900, Saudi Arabia; 3 The University of Western Australia, Oceans Institute, 35 Stirling Hwy, Crawley, WA 6009, Australia

## Abstract

China is facing intense coastal eutrophication. Large-scale seaweed aquaculture in China is popular, now accounting for over 2/3’s of global production. Here, we estimate the nutrient removal capability of large-scale Chinese seaweed farms to determine its significance in mitigating eutrophication. We combined estimates of yield and nutrient concentration of Chinese seaweed aquaculture to quantify that one hectare of seaweed aquaculture removes the equivalent nutrient inputs entering 17.8 ha for nitrogen and 126.7 ha for phosphorus of Chinese coastal waters, respectively. Chinese seaweed aquaculture annually removes approximately 75,000 t nitrogen and 9,500 t phosphorus. Whereas removal of the total N inputs to Chinese coastal waters requires a seaweed farming area 17 times larger than the extant area, one and a half times more of the seaweed area would be able to remove close to 100% of the P inputs. With the current growth rate of seaweed aquaculture, we project this industry will remove 100% of the current phosphorus inputs to Chinese coastal waters by 2026. Hence, seaweed aquaculture already plays a hitherto unrealized role in mitigating coastal eutrophication, a role that may be greatly expanded with future growth of seaweed aquaculture.

High population density, intensive agriculture and industrial activities have led to a huge increase in nutrient inputs to Chinese coastal waters^1^,^2^,^3^,^4^,^5^, which receive some the world’s highest riverine and atmospheric inputs of nutrients to the coastal ocean^6^,^7^. Nutrient inputs to Chinese coastal waters are dominated by non-point sources, largely derived from excessive fertilizer application^3^,^8^,^9^, while point sources, easier to control, represent less than 15% of nutrient inputs^3^,^8^. In addition, assessment of nutrient inputs to the Yellow Sea of China identified atmospheric deposition as the main vector for nutrient inputs to the coastal ocean, supplying twice the nutrient inputs delivered by rivers^10^. The consequences are widespread eutrophication, manifested in an increased frequency of harmful algal blooms^11^,^12^ and widespread hypoxia^4^,^13^ across Chinese coastal waters. Hence, nutrient pollution leading to coastal eutrophication is recognized as one of the most significant environmental problems, posing a great challenge to China^1^,^3^,^12^.

The rise of seaweed aquaculture represents a parallel major development in Chinese coastal waters^14^,^15^, with seaweed aquaculture production rising 7.7 fold from 259,839 t dry weight (DW) in 1978 to 2,004,600 t DW in 2014^16^, growing at 7.96% year^−1^ at present (Fig. 1). China dominates seaweed aquaculture production worldwide, contributing over 2/3’s of global production^15^,^17^. This production is mainly comprised of *Saccharina japonica* and *Gracilariopsis spp.*^18^, with other species accounting for 17.3% of production^16^. Individual seaweed farms have reached such an extent as to be visible from space, with individual farms spanning more than 35 km^2^ along the coastal regions of China^14^ (Fig. 2).

The scale of seaweed aquaculture in China is such that it may be of regional biogeochemical significance^14^,^15^,^17^,^19^. In particular, seaweed take up dissolved nutrients, which are then removed from coastal waters to land after harvest^19^,^20^,^21^. Indeed, it has been hypothesized that macroalgal production helps remove excess nutrients and replenish oxygen in water, thereby alleviating the effects of eutrophication on coastal ecosystems^18^,^19^,^22^,^23^,^24^. Yet, the role of seaweed aquaculture as a vector for the removal of nutrients from Chinese coastal waters has not yet been evaluated. Here we demonstrate that Chinese seaweed aquaculture plays a significant role in removing nutrients from Chinese coastal waters. We do so by first providing a first order estimate of the magnitude of nutrient removal by Chinese seaweed aquaculture and then assessing its significance by comparing the magnitude of nutrient removal, per unit area and across the entire Chinese coastal zone, with that of nutrient inputs.

## Results and Discussion

**Figure 1:**
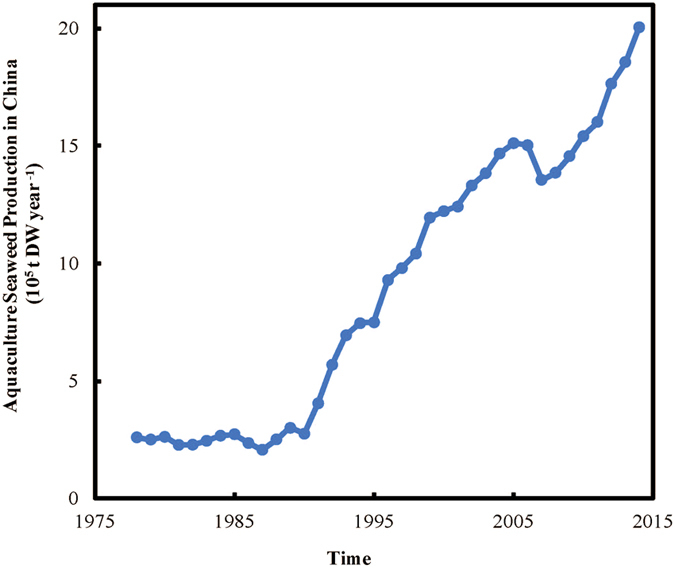
Timeline of aquaculture seaweed production in China (1978–2014). Data Source: China Fishery Statistical Yearbook from 1979 to 2015^30^. The production reported before 2003 includes wild collection of seaweed, which is about 2% of the aquaculture production, on average.

The magnitude of nutrient removal by Chinese seaweed aquaculture is dependent on the yield and the nutrient concentration in the seaweed tissues, which depends, in turn, on the species composition. Seaweed production reached two million t DW in 2014^16^, growing at 7.96% year^−1^ at present (Fig. 1). Provided a mean (±SE) reported nitrogen (N) and phosphorus (P) concentration for the dominant species in Chinese aquaculture of 3.71 ± 1.15N % DW and 0.52 ± 0.23P % DW for *Saccharina japonica*, 4.53 ± 0.43N % DW and 0.34 ± 0.05P % DW for *Gracilariopsis spp.*, and an estimated average concentration of 3.52 ± 0.40N % DW and 0.38 ± 0.14P % DW for the rest of the species (Table 1), the calculated N and P removal with seaweed aquaculture in 2014 was substantial, at 75,371 ± 18,424 t N and 9,496 ± 3,875 t P, respectively.

Seaweed aquaculture in China is characterised by a yield of about 1,604 t DW km^−2^ 16, corresponding, using the estimated average tissue N and P concentration in seaweed aquaculture provided above, to a removal of 60.31 t N and 7.60 t P km^−2^ seaweed farm per year (Table 1). Nitrogen and phosphorus inputs are largely derived in inorganic form, but include a small fraction of dissolved organic nitrogen and, even small, dissolved organic phosphorus^25^. However, seaweed have been shown to be able to take up low-molecular forms of dissolved organic nitrogen as well^26^,^27^. The calculations of nutrient removal by seaweed aquaculture in China presented above involve no assumptions as to the form of nitrogen or phosphorus supporting the uptake. Resolving regional variability in nutrient inputs and seaweed production would require additional information that is currently unavailable. We hope that the calculations above will provide a stimulus to generate the data required for regional analysis and to examine directly the uptake by seaweed of nutrient deposited from the atmosphere.

The estimates provided above confirm the important contribution of Chinese seaweed aquaculture to nutrient removal in China. This capacity is further confirmed by reports of reductions in dissolved inorganic nutrient concentrations in Chinese seaweed farms. Published case-studies assessing nutrient removal in four seaweed farms in China indicate that the nutrient concentrations in seawater decreased by, on average (±SE), 53% ± 7%, 47% ± 10% and 47% ± 3% for NH_4_-N, NO_3_-N and PO_4_-P, respectively, within these seaweed farms compared to that in nearby non-cultivation coastal waters (Table 2). Total N and P removal by seaweed aquaculture represents 5.56% and 39.60% of the total estimated input of N and P to Chinese coastal waters of 1,350,053 t N and 23,982 t P, respectively, with the N removed with seaweed aquaculture representing about 66% of the total point source inputs to Chinese coastal waters^3^,^8^. This nutrient removal capacity is remarkable, provided that seaweed aquaculture occupies only about 1,250 km^2^ of coastal area. This represents only approximately 0.3% of the area of Chinese territorial sea waters^28^, suggesting that there is ample scope to continue to increase the seaweed aquaculture area further. Indeed, we calculate that one km^2^ of seaweed farm removes the annual N and P inputs received by 17.8 km^2^ and 126.7 km^2^ of Chinese coastal waters, respectively (Table 1), providing an estimate of the nutrient removal footprint area of Chinese seaweed farms.

The seaweed area required to remove N and P equivalent to the 100% of the present N and P inputs to Chinese coastal waters can be calculated, as the ratio of the total input estimate provided above and the removal rate by seaweed farms per km^2^, at 22,470 km^2^ for N and 3,155 km^2^ for P, respectively. Whereas removal of the total N inputs to Chinese coastal waters requires a seaweed farming area 17 times larger than the extant area, one and a half times more of the seaweed area would be able to remove close to 100% of the P inputs. At the current annual rate of 7.96% increase in seaweed aquaculture^16^, the area required to remove 100% of the P inputs will be met by 2026. Although fast urbanization in China may still enhance inputs of nutrients to coastal area in the following decades^1^,^29^, causing further increase of total riverine N and P inputs by 30–200% to 2050^29^, the rapidly developing seaweed aquaculture would have the capacity to remove almost all the riverine inputs of P and much of N inputs. Hence, the efficiency of seaweed farming in removing nutrients from coastal waters may lead to phosphorus limitation in the future, even under the large nutrient inputs to Chinese coastal waters, thereby preventing the proliferation of dense algal blooms. These are, however, theoretical calculations, as neither nutrient inputs nor seaweed farms are, or will be, homogeneously distributed along the coast. Moreover, phytoplankton and wild and aquaculture seaweed will compete for nutrient inputs, particularly as further growth of seaweed aquaculture results in reduced nutrient concentrations in Chinese coastal waters. The much larger biomass density (g DW m^−3^) in seaweed farms compared to phytoplankton and the fact that seaweed biomass is typically near the surface in aquaculture farms suggest that seaweed might be able to take up the bulk of the atmospheric nutrient deposition in farmed areas, an important component of the nitrogen input to Chinese coastal waters.

Nutrient concentrations are expected to decline with increasing expansion of seaweed farming, to the extent that seaweed aquaculture may in the future become nutrient limited, particularly in areas supporting large seaweed farms and moderate or low nutrient inputs. Seaweed aquaculture may overcome future nutrient limitation by integrating it with animal aquaculture in polyculture, thereby using the nutrient emissions from animal metabolism and excess feed to maximize economic, environmental and ecological benefits^18^. Nevertheless, the annual growth rate of 7.96% can only be sustained if farming continues to be economically profitable in China, which requires a parallel rise in the domestic and worldwide demands for seaweed products. This requires an increase in per capita seaweed consumption, diversification of outputs to other industries, as well as economic compensation, possibly as tax relief or direct subsidies, to seaweed farmers for the benefits of the activity in terms of nutrient removal.

In conclusion, we demonstrate here that seaweed aquaculture has reached a scale in China where it is already delivering considerable environmental benefits in terms of nutrient removal from coastal waters. Further growth of seaweed aquaculture can play a pivotal role, along with sustained efforts to reduce nutrient inputs, in tackling the hitherto mounting problem of coastal eutrophication in China.

## Methods

We extracted aquaculture seaweed production and percentage of major seaweed cultivation from annual government fishery reports in China for 37 years^30^ from 1978 to 2015 (Fig. 1). Then, we obtained the current Chinese seaweed yield (t DW km^−2^) by dividing the 2014 production by the 2014 seaweed aquaculture area, both reported in June, 2015^16^.

We used published estimates of tissue N and P concentration of Chinese seaweed aquaculture species based on a search on Web of Science^®^ and CNKI^®^ accessed until December 2015. The search using “nutrient concentration”, “China” and “seaweed” as keywords yielded a total of 22 papers reporting nutrient concentration of seaweed species in China. From these papers, we selected those corresponding to the seven main targeted species of seaweed aquaculture in China (Tables S2–1 and S2–2), and calculated their mean (±SE) nutrient concentration. We then combined the average tissue nutrient concentration with the contribution of each species to aquaculture production in China (in 2014) to yield a weighted-average nutrient concentration in seaweed aquaculture in China (Tables S2–3). We combined the weighted-average nutrient concentration with the average yield of seaweed aquaculture in China to calculate nutrient removal per km^2^ for Chinese coastal areas.

Estimates of nutrient inputs are not available for the entire Chinese territorial sea waters, but detailed budgets have been reported for the Yellow Sea^10^ and East China Sea^31^, including both nutrient inputs with riverine discharge and atmospheric inputs. These were used to calculate the total input of N and P to the 0.4 million km^2^ of Chinese territorial sea waters^28^ where seaweed aquaculture is based. We first calculated the flux of N and P per km^2^ for the Yellow Sea, which was reported as the yearly nutrient input from riverine and atmospheric sources^10^, by diving the total input by the area of this ecosystem. For the East China Sea, the nutrient input, including riverine discharge and atmospheric inputs, was reported for each of two seasons^31^. We then divided the average input by the area of this ecosystem to obtain the flux of N and P per km^2^. We then combined the two estimates for the Yellow Sea and East China Sea as a weighted-average flux to obtain a characteristic nutrient flux per km^2^ (t yr^−1^ km^−2^), to Chinese coastal waters, and multiplied this estimate by the 0.4 million km^2^ of Chinese territorial sea waters^28^ to obtain the total nutrient input to Chinese coastal waters. The estimates obtained were consistent with an independent estimates of the total nutrient input to the East China Sea^32^. These estimates of nutrient inputs are likely to produce an upper estimate of fluxes, and therefore a lower estimate of removal with seaweed aquaculture, as the Yellow Sea and East China Seas receive particularly high nutrient inputs^10^,^31^. All statistical analyses were conducted using JMP v10 software.

## Additional Information

**How to cite this article**: Xiao, X. *et al*. Nutrient removal from Chinese coastal waters by large-scale seaweed aquaculture. *Sci. Rep.* **7**, 46613; doi: 10.1038/srep46613 (2017).

**Publisher's note:** Springer Nature remains neutral with regard to jurisdictional claims in published maps and institutional affiliations.

## Supplementary Material

Supplementary Information

## Figures and Tables

**Figure 2:**
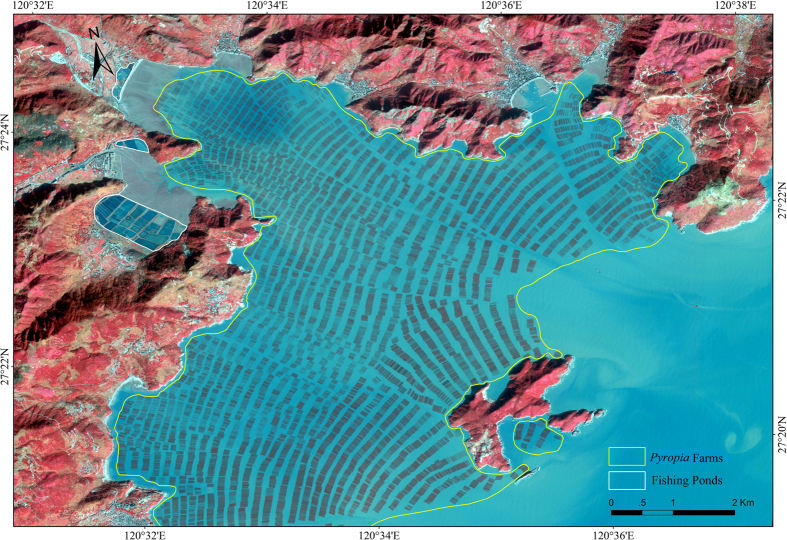
Satellite Gao-Fen 2 image of the coastal area of Cang-nan county, Zhejiang province, southeastern China, showing large-scale seaweed (*Pyropia*) aquaculture farms. SuperMap GIS 8 C (http://www.supermap.com/xhtml/SuperMap-GIS-8C.html) was used to generate this map.

**Table 1:** Total nutrient removal by seaweed aquaculture in China and the nutrient removal capacity of Chinese seaweed farms km^−2^.

**Total for China (2014)**		
Seaweed Production^16^	2.00	million t DW
Seaweed Area^16^	1,250	km^2^
N concentration*	3.76 ± 0.92	% DW
P concentration*	0.47 ± 0.19	% DW
N removal	75,371 ± 18,423	t N year^−1^
P removal	9,496 ± 3,875	t P year^−1^
**Per km**^**2**^ **of Seaweed Farm and Year**		
Seaweed Production	1,604	t DW
N concentration*	3.76 ± 0.92	% DW
P concentration*	0.47 ± 0.19	% DW
N removal	60.31	t N km^−2^ year^−1^
P removal	7.60	t P km^−2^ year^−1^
N input**	3.38	t N km^−2^ year^−1^
P input**	0.06	t P km^−2^ year^−1^
Seaweed Farm N Footprint area	17.8	km^2^ of coastal ocean removed of N inputs km^−2^ of seaweed farm
Seaweed Farm P Footprint area	126.7	km^2^ of coastal ocean removed of P inputs km^−2^ of seaweed farm

The seaweed farm N and P footprint area refers to the km^2^ of Chinese coastal waters receiving nutrient inputs equivalent to those removed by one km^2^ of seaweed farms. *The average tissue nutrient concentrations of Chinese seaweed, as weighted per species (Tables S2–3); **Nutrient input from the inventory integrating the riverine and atmosphere resources, weighted by area of East China Sea and Yellow Sea^10^,^31^.

**Table 2:** Summary of case studies on nutrient removal by seaweed aquaculture in China, showing local biogeochemical significance of seaweed farms.

	Location			Seaweed Cultivation			Seawater Nutrient Concentration Reduction Efficiency			Ref
	Bay	Region	Latitude	Year	Area (km^2^)	Species	NH_4_-N (%)	NO_3_-N (%)	PO_4_-P (%)	
1	Radial sandbank, Jiangsu	South Yellow Sea	120°′30′–121°40′E, 31°30′–33°40′N	2012–2013	N/A	*Pyropia yezoensis*	44%	49%	45%	Wu *et al*.^20^
2	Xiangshan harbor, Zhejiang	East China Sea	121°36′–121°37′ E, 29°32′ N	2011	0.000054 (Cage study)	*Gracilariopsis longissima*	22–61%	24–47%	22–58%	Huo *et al*.^21^
3	Hangzhou Bay, Shanghai	East China Sea	121°18′ E, 30°42′ N	2006–2007	0.075	*Gracilariopsis longissima*	54%	75%	49%	Huo *et al*.^33^
4	Lusi coast, Jiangsu	East China Sea	121°35′ E, 32°05′ N	2002–2004	3.00	*Pyropia yezoensis*	50–94%	21–38%	42–67%	He *et al*.^22^
**Average**							53% ± 7%	47% ± 10%	47% ± 3%	

## References

[b1] Qu H. J. & Kroeze C. Nutrient export by rivers to the coastal waters of China: management strategies and future trends. Reg. Environ. Change 12, 153–167 (2012).

[b2] Kim T. W., Lee K., Najjar R. G., Jeong H. D. & Jeong H. J. Increasing N abundance in the Northwestern Pacific Ocean due to atmospheric nitrogen deposition. Science 334, 505–509 (2011). 21940860 10.1126/science.1206583

[b3] Yan W., Mayorga E., Li X., Seitzinger S. P. & Bouwman A. Increasing anthropogenic nitrogen inputs and riverine DIN exports from the Changjiang River basin under changing human pressures. Global Biogeochem. Cy. 24, GB0A06 (2010).

[b4] Cai W. J. . Acidification of subsurface coastal waters enhanced by eutrophication. Nat. Geosci. 4, 766–770 (2011).

[b5] Gu B. . Rapid growth of industrial nitrogen fluxes in China: Driving forces and consequences. Science China Earth Sciences 56, 662–670 (2013).

[b6] Tysmans D. J., Löhr A. J., Kroeze C., Ivens W. P. & van Wijnen J. Spatial and temporal variability of nutrient retention in river basins: a global inventory. Ecol. Indicators 34, 607–615 (2013).

[b7] Boyer E. W. . Riverine nitrogen export from the continents to the coasts. Global Biogeochem. Cy. 20, GB1S91 (2006).

[b8] Ongley E. D., Zhang X. & Yu T. Current status of agricultural and rural non-point source pollution assessment in China. Environ. Pollut. 158, 1159–1168 (2010). 19931958 10.1016/j.envpol.2009.10.047

[b9] Sun B. . Agricultural non-point source pollution in China: causes and mitigation measures. Ambio 41, 370–379 (2012). 22311715 10.1007/s13280-012-0249-6 PMC3393061

[b10] Liu S. . Inventory of nutrient compounds in the Yellow Sea. Cont. Shelf Res. 23, 1161–1174 (2003).

[b11] Yan T., Zhou M. & Zou J. A national report on harmful algal blooms in China. Harmful algal blooms in the PICES region of the North Pacific. 21 (2002).

[b12] Liu D. . The world’s largest macroalgal bloom in the Yellow Sea, China: formation and implications. Estuar. Coast. Shelf Sci. 129, 2–10 (2013).

[b13] Zhu Z. . Hypoxia off the Changjiang (Yangtze River) Estuary: oxygen depletion and organic matter decomposition. Mar. Chem. 125, 108–116 (2011).

[b14] Duarte C. M. . Will the oceans help feed humanity? Bioscience 59, 967–976 (2009).

[b15] Mazarrasa I., Olsen Y. S., Mayol E., Marbà N. & Duarte C. M. Rapid growth of seaweed biotechnology provides opportunities for developing nations. Nat. Biotechnol. 31, 591–592 (2013). 23839140 10.1038/nbt.2636

[b16] China Fishery Statistical Yearbook. (China Agriculture Press, 2015).

[b17] Mazarrasa I., Olsen Y. S., Mayol E., Marbà N. & Duarte C. M. Global unbalance in seaweed production, research effort and biotechnology markets. Biotechnol. Adv. 32, 1028–1036 (2014). 24858315 10.1016/j.biotechadv.2014.05.002

[b18] Shi H., Zheng W., Zhang X., Zhu M. & Ding D. Ecological–economic assessment of monoculture and integrated multi-trophic aquaculture in Sanggou Bay of China. Aquaculture 410, 172–178 (2013).

[b19] Fei X. Solving the coastal eutrophication problem by large scale seaweed cultivation. Hydrobiologia 512, 145–151 (2004).

[b20] Wu H. . Bioremediation efficiency of the largest scale artificial *Porphyra yezoensis* cultivation in the open sea in China. Marine Pollution Bulletin 95, 289–296 (2015). 25865344 10.1016/j.marpolbul.2015.03.028

[b21] Huo Y. . Bioremediation efficiency of *Gracilaria verrucosa* for an integrated multi-trophic aquaculture system with *Pseudosciaena crocea* in Xiangshan harbor, China. Aquaculture 326, 99–105, doi: 10.1016/j.aquaculture.2011.11.002 (2012).

[b22] He P. . Bioremediation efficiency in the removal of dissolved inorganic nutrients by the red seaweed, *Porphyra yezoensis*, cultivated in the open sea. Water Research 42, 1281–1289 (2008). 17959220 10.1016/j.watres.2007.09.023

[b23] Jiang Z. J., Fang J. G., Mao Y. Z. & Wang W. Eutrophication assessment and bioremediation strategy in a marine fish cage culture area in Nansha Bay, China. J. Appl. Phycol. 22, 421–426 (2010).

[b24] Yu Z., Zhu X., Jiang Y., Luo P. & Hu C. Bioremediation and fodder potentials of two *Sargassum spp.* in coastal waters of Shenzhen, South China. Marine Pollution Bulletin 85, 797–802, doi: 10.1016/j.marpolbul.2013.11.018 (2014). 24332756

[b25] Shen Z. Phosphorus and Silicate Fluxes in the Yangtze River. Acta Geographica Sinica 61, 741–751 (2006).

[b26] Julia C. P. & Hurd C. L. Kinetics of nitrate, ammonium, and urea uptake by four intertidal seaweeds from New Zealand. J. Phycol. 40, 534–545 (2004).

[b27] Tyler A. C., Mcglathery K. J. & Macko S. A. Uptake of urea and amino acids by the macroalgae *Ulva lactuca* (Chlorophyta) and *Gracilaria vermiculophylla* (Rhodophyta). Mar. Ecol. Prog. Ser. 294, 161–172 (2005).

[b28] Luo X. . Chinese coastal seas are facing heavy atmospheric nitrogen deposition. Environ. Res. Lett. 9, 095007 (2014).

[b29] Strokal M. . Increasing eutrophication in the coastal seas of China from 1970 to 2050. Mar. Pollut. Bull. 85, 123–140 (2014). 24981103 10.1016/j.marpolbul.2014.06.011

[b30] China Fishery Statistical Yearbook. (China Agriculture Press, 1979–2015).

[b31] Zhang J., Liu S., Ren J., Wu Y. & Zhang G. Nutrient gradients from the eutrophic Changjiang (Yangtze River) Estuary to the oligotrophic Kuroshio waters and re-evaluation of budgets for the East China Sea Shelf. Prog. Oceanogr. 74, 449–478 (2007).

[b32] Chen C.-T. A. Distributions of nutrients in the East China Sea and the South China Sea connection. Journal of Oceanography 64, 737–751, doi: 10.1007/s10872-008-0062-9 (2008).

[b33] Huo Y. Z. . Bioremediation efficiencies of *Gracilaria verrucosa* cultivated in an enclosed sea area of Hangzhou Bay, China. Journal of Applied Phycology 23, 173–182, doi: 10.1007/s10811-010-9584-9 (2011).

