# Supplementary material for: Nutrient removal from Chinese coastal waters by large-scale seaweed aquaculture
Source: Sci Rep. 2017 Apr 21;7:46613. doi: 10.1038/srep46613 (PMC5399451; doi:10.1038/srep46613)
Supplement: Supplementary Information [file srep46613-s1.doc]

**Supplementary Information**

**Nutrient removal from Chinese coastal waters**

**by large-scale seaweed aquaculture**

Xi Xiao^a^, Susana Agusti^b^, Fang Lin^a^, Ke Li^a^, Yaoru Pan^a^, Yan Yu^a^, Yuhan Zheng^a^, Jiaping Wu^a,*^, Carlos M. Duarte^b,c, *^

^a^ Ocean College, Zhejiang University, 1 Zheda Road, Zhoushan, Zhejiang 316021, China

^b^ Red Sea Research Center, King Abdullah University of Science and Technology, Thuwal 23955-6900, Saudi Arabia

^c^ Oceans Institute, the University of Western Australia, 35 Stirling Hwy, Crawley, WA 6009, Australia

^*^ To whom correspondence may be addressed. Email: jw67@zju.edu.cn or carlos.duarte@kaust.edu.sa

**Table of Content**

Section 1

Table S1. Nutrients input into the Chinese seas

Section 2

Table S2-1. The tissue N concentration of Chinese seaweed aquaculture

Table S2-2. The tissue P concentration of Chinese seaweed aquaculture

Table S2-3. Weighted average values of tissue N and P concentration for seaweed aquaculture in China

Table S2-4. Species names of seaweed aquaculture in China from references and their current accepted names from AlgaeBase

**Section 1.**

We converted the published nutrient inventories of Chinese seas into nutrient flux (t yr^-1^ km^-2^) (Table S1), and then multiply the weighted average nutrient input flux by the area of Chinese coastal seas (total area approx. 0.4 million km^2^). Thus we estimated nutrient imported to Chinese coastal waters from both riverine and atmosphere resources, as well as the seaweed farm N and P footprints.

**Table S1. Nutrients input into the Chinese seas.**

|  |  | **Nutrient discharge**  **(10^9^ mol year^-1^)** | | |  | **Input (t km^2^ year^-1^)** | | |
| --- | --- | --- | --- | --- | --- | --- | --- | --- |
|  |  | Riverine | Atmosphere | Total |  | Riverine | Atmosphere | Total |
| *Yellow Sea^*^* | NO_3_-N | 24.000 | 12.400 | 36.400 |  | 0.884 | 0.457 | 1.341 |
|  | NH_4_-N | 2.700 | 37.000 | 39.700 |  | 0.099 | 1.363 | 1.463 |
|  | N_sum_ | 26.700 | 49.400 | 76.100 |  | 0.984 | 1.820 | 2.804 |
|  | PO_4_-P | 0.420 | 0.890 | 1.310 |  | 0.034 | 0.073 | 0.107 |
| *East China Sea^**^* | NO_3_-N | 89.408 | 17.094 | 106.502 |  | 2.276 | 0.435 | 2.711 |
|  | NH_4_-N | 9.063 | 32.540 | 41.603 |  | 0.231 | 0.828 | 1.059 |
|  | N_sum_ | 98.471 | 49.634 | 148.105 |  | 2.507 | 1.263 | 3.770 |
|  | PO_4_-P | 0.420 | 0.069 | 0.489 |  | 0.024 | 0.004 | 0.028 |

*^*^*Liu, S. *et al.* Inventory of nutrient compounds in the Yellow Sea. *Continental Shelf Research* **23**, 1161-1174 (2003).

*^**^* Zhang, J., Liu, S., Ren, J., Wu, Y. & Zhang, G. Nutrient gradients from the eutrophic Changjiang (Yangtze River) Estuary to the oligotrophic Kuroshio waters and re-evaluation of budgets for the East China Sea Shelf. *Progress in Oceanography* **74**, 449-478 (2007).

**Section 2.**

We used the published experimental data on the tissue N and P concentration of Chinese seaweed production based on a search on Web of Science® and CNKI® accessed until December 2015 (Supplementary Information). Combining the tissue nutrient contents and production percentages for each major seaweed species, we further calculated the annual nutrient removal by seaweed aquaculture in China (Table S2-1, Table S2-2, Table S2-3).

**Table S2-1. Tissue N concentration reported for Chinese seaweed aquaculture. Species name updated relative to that in source to display currently accepted name (see equivalence in Table S2-4).**

| **Country** | **Species** | **Tissue N conc. Mean (% DW)** | **Reference *** |
| --- | --- | --- | --- |
| China | *Saccharina japonica* | 3.22 | 1 |
| China | *Saccharina japonica* | 2.04 | 2 |
| China | *Saccharina japonica* | 8.87 | 3 |
| China | *Saccharina japonica* | 4.82 | 4 |
| China | *Saccharina japonica* | 1.43 | 5 |
| China | *Saccharina japonica* | 1.88 | 6 |
| China | *Pyropia yezoensis* | 6.03 | 7 |
| China | *Pyropia yezoensis* | 6.03 | 7 |
| China | *Pyropia yezoensis* | 2.09 | 8 |
| China | *Pyropia yezoensis* | 3.07 | 8 |
| China | *Pyropia yezoensis* | 4.72 | 8 |
| China | *Pyropia yezoensis* | 4.71 | 8 |
| China | *Pyropia yezoensis* | 3.81 | 8 |
| China | *Pyropia yezoensis* | 4.50 | 9 |
| China | *Pyropia yezoensis* | 5.45 | 9 |
| China | *Pyropia yezoensis* | 3.56 | 9 |
| China | *Pyropia yezoensis* | 6.26 | 9 |
| China | *Pyropia yezoensis* | 6.84 | 10 |
| China | *Gracilariopsis lemaneiformis* | 2.58 | 11 |
| China | *Gracilariopsis lemaneiformis* | 3.14 | 11 |
| China | *Gracilariopsis lemaneiformis* | 3.61 | 12 |
| China | *Gracilariopsis lemaneiformis* | 3.65 | 11 |
| China | *Gracilariopsis lemaneiformis* | 3.84 | 12 |
| China | *Gracilariopsis lemaneiformis* | 3.94 | 12 |
| China | *Gracilariopsis lemaneiformis* | 4.07 | 12 |
| China | *Gracilariopsis longissima* | 4.80 | 13 |
| China | *Gracilariopsis lemaneiformis* | 4.88 | 14 |
| China | *Gracilariopsis lemaneiformis* | 5.01 | 14 |
| China | *Gracilariopsis lemaneiformis* | 5.15 | 14 |
| China | *Gracilariopsis lemaneiformis* | 5.39 | 14 |
| China | *Gracilariopsis lemaneiformis* | 8.88 | 11 |
| China | *Kappaphycus striatus* | 0.46 | 6 |
| China | *Sargassum hemiphyllum* | 1.86 | 15 |
| China | *Sargassum piluliferum* | 2.00 | 15 |
| China | *Sargassum piluliferum* | 2.11 | 15 |
| China | *Sargassum hemiphyllum* | 2.29 | 15 |
| China | *Sargassum graminifolium* | 4.08 | 4 |
| China | *Ulva australis* | 2.78 | 16 |
| China | *Ulva australis* | 3.05 | 16 |
| China | *Ulva australis* | 3.68 | 16 |
| China | *Ulva australis* | 3.65 | 16 |
| China | *Ulva australis* | 4.01 | 16 |
| China | *Ulva australis* | 4.05 | 16 |
| China | *Ulva australis* | 3.70 | 16 |
| China | *Ulva australis* | 3.55 | 16 |
| China | *Ulva australis* | 5.46 | 4 |
| China | *Ulva australis* | 2.29 | 6 |
| China | *Ulva fasciata* | 1.77 | 6 |
| Japan | *Undaria pinnatifida* | 3.08 | 17 |
| Japan | *Undaria pinnatifida* | 3.04 | 17 |
| Japan | *Undaria pinnatifida* | 3.69 | 17 |
| Japan | *Undaria pinnatifida* | 2.09 | 18 |
| Japan | *Undaria pinnatifida* | 1.57 | 18 |

**Table S2-2. Tissue P concentration reported for Chinese seaweed aquaculture species.** Species name updated relative to that in source to display currently accepted name (see equivalence in Table S2-4).

| **Country** | **Species** | **Tissue P Conc.  Mean (% DW)** | **Reference *** |
| --- | --- | --- | --- |
| China | *Saccharina japonica* | 1.68 | 3 |
| China | *Saccharina japonica* | 0.32 | 4 |
| China | *Saccharina japonica* | 0.32 | 19 |
| China | *Saccharina japonica* | 0.43 | 19 |
| China | *Saccharina japonica* | 0.19 | 20 |
| China | *Saccharina japonica* | 0.19 | 21 |
| China | *Undaria spp.* | 0.15 | 19 |
| China | *Undaria spp.* | 0.27 | 19 |
| China | *Undaria pinnatifida* | 0.02 | 20 |
| China | *Undaria pinnatifida* | 0.02 | 21 |
| China | *Pyropia yezoensis* | 1.02 | 7 |
| China | *Pyropia yezoensis* | 1.02 | 7 |
| China | *Pyropia yezoensis* | 0.18 | 10 |
| China | *Gracilariopsis lemaneiformis* | 0.19 | 11 |
| China | *Gracilariopsis lemaneiformis* | 0.23 | 11 |
| China | *Gracilariopsis lemaneiformis* | 0.33 | 14 |
| China | *Gracilariopsis lemaneiformis* | 0.34 | 11 |
| China | *Gracilariopsis lemaneiformis* | 0.51 | 11 |
| China | *Gracilariopsis lemaneiformis* | 0.47 | 11 |
| China | *Sargassum hemiphyllum* | 0.11 | 15 |
| China | *Sargassum hemiphyllum* | 0.13 | 15 |
| China | *Sargassum piluliferum* | 0.14 | 15 |
| China | *Sargassum piluliferum* | 0.17 | 15 |
| China | *Sargassum fusiforme* | 0.11 | Dongtou island, this study* |
| China | *Sargassum graminifolium* | 0.36 | 4 |
| China | *Sargassum fusiforme* | 0.12 | 20 |
| China | *Sargassum fusiforme* | 0.12 | 21 |
| China | *Ulva sp.* | 0.08 | Dongtou island, this study* |
| China | *Ulva australis* | 0.13 | 20 |
| China | *Ulva australis* | 0.10 | 20 |
| China | *Ulva australis* | 0.13 | 21 |
| China | *Ulva australis* | 0.10 | 21 |
| China | *Ulva australis* | 0.40 | 4 |
| China | *Kappaphycus striatus* | 4.87 | 22 |
| China | *Kappaphycus striatus* | 3.31 | 22 |

* These species were collected from the seaweed farm in Dongtou island (121°11′06′′ E, 27°51′42′′ N), Zhejiang, China, and analyzed by the authors.

**Table S2-3. Weighted average values of tissue N and P concentration for seaweed aquaculture in China**

|  | **Tissue N conc.** | |  |  | **Tissue P conc.** | |  |  | **Contribution to aquaculture production (%)** |
| --- | --- | --- | --- | --- | --- | --- | --- | --- | --- |
|  | **(% DW)** | |  |  | **(% DW)** | |  |  |
|  | Mean | SE | n^*^ |  | Mean | SE | n^*^ |  |
| *Saccharina* | 3.71 | 1.15 | 6 |  | 0.52 | 0.23 | 6 |  | 69.35 |
| *Undaria* | 2.69 | 0.38 | 5 |  | 0.12 | 0.06 | 4 |  | 10.35 |
| *Pyropia* | 4.76 | 0.41 | 12 |  | 0.74 | 0.28 | 3 |  | 5.82 |
| *Gracilariopsis* | 4.53 | 0.43 | 13 |  | 0.34 | 0.05 | 6 |  | 13.36 |
| *Kappaphycus* | 0.46 | 0.00 | 1 |  | 4.09 | 0.78 | 2 |  | 0.22 |
| *Sargassum* | 2.47 | 0.41 | 5 |  | 0.16 | 0.03 | 8 |  | 0.89 |
| *Ulva* | 3.45 | 0.30 | 11 |  | 0.16 | 0.05 | 6 |  | 0.01 |
| **Weighted Average** | 3.76 | 0.92 |  |  | 0.47 | 0.19 |  |  |  |

^*^ n represents the number of assessments.

**Table S2-4. Species names of seaweed aquaculture in China from references and their current accepted names from AlgaeBase**

| **Name used in sources for Tables S2-1 and S2-2** | **Current accepted name (from AlgaeBase*)** |
| --- | --- |
| *Laminaria japonica* | *Saccharina japonica* |
| *Gracilaria lemaneiformes* | *Gracilariopsis lemaneiformis* |
| *Porphyra yezoensis* | *Pyropia yezoensis* |
| *Gracilaria verrucosa* | *Gracilariopsis longissima* |
| *Eucheuma striatum* | *Kappaphycus striatus* |
| *Sargassum henslowianum* | *Sargassum piluliferum* |
| *Ulva pertusa* | *Ulva australis* |

* Species search link in AlgaeBase: http://www.algaebase.org/search/species/

**References for Section 2**

1 Shanshan, L. Study on the change of morphology and composition of kelp and *Microcystis aeruginosa* in microbia conversion process, Anhui University, (2014).

2 Rongjun, W. *et al.* Nitrogen requirement of growth and photosynthesis in the juvenile sporophyte of *Laminaria japonica*. *Marine Science Bulletin*, 36-42 (2006).

3 Shufen, S. *Bioremediation of Laminaria japonica and the establishment of its gametophyte clones*, Fujian Normal University, (2013).

4 Jiandao, H., Xiaoping, H. & Weizhong, Y. Contents of TN, TP in macroalgal and its significance for remediation of coastal environment. *Journal of Oceanography in Taiwan Strait*, 316-321 (2005).

5 Zhang, J. *et al.* Growth and loss of mariculture kelp *Saccharina japonica* in Sungo Bay, China. *Journal of Applied Phycology* **24**, 1209-1216 (2012).

6 Xiao, F., Lijun, L., Tiancheng, Z., Qingxiang, L. & Yanxia, Z. Chemical composition of economic seaweeds from the coast of China. *Qceanologia and Limnologia Sinica*, 199-207 (1995).

7 He, P. *et al.* Bioremediation efficiency in the removal of dissolved inorganic nutrients by the red seaweed, *Porphyra yezoensis*, cultivated in the open sea. *Water Research* **42**, 1281-1289 (2008).

8 Wu, C. Y. *et al.* Utilization of ammonium-nitrogen by *Porphyra yezoensis* and *Gracilaria verrucosa*. *Hydrobiologia* **116**, 475-477 (1984).

9 Xinshu, L., Guanghui, F., Juntian, X. & Peimin, H. Effect of nitrogen and phosphorus enrichment on growth and biochemical composition of laver *Porphyra yezoensis*. *Fisheries Science*, 544-548 (2012).

10 Wu, H. *et al.* Bioremediation efficiency of the largest scale artificial *Porphyra yezoensis* cultivation in the open sea in China. *Marine Pollution Bulletin* **95**, 289-296 (2015).

11 Peng, C.-L. *et al.* Response of *Gracilaria lemaneiformis* to nitrogen and phosphorus eutrophic seawater. *Zhiwu Shengtai Xuebao* **31**, 505-512 (2007).

12 Mao, Y. et al. Potential of the seaweed Gracilaria lemaneiformis for integrated multi-trophic aquaculture with scallop Chlamys farreri in North China. J Appl Phycol 21: 649 (2009).

13 Huo, Y. *et al.* Bioremediation efficiency of *Gracilaria verrucosa* for an integrated multi-trophic aquaculture system with *Pseudosciaena crocea* in Xiangshan harbor, China. *Aquaculture* **326**, 99-105, doi:10.1016/j.aquaculture.2011.11.002 (2012).

14 Zhou, Y. *et al.* Bioremediation potential of the macroalga *Gracilaria lemaneiformis* (Rhodophyta) integrated into fed fish culture in coastal waters of north China. *Aquaculture* **252**, 264-276, doi:10.1016/j.aquaculture.2005.06.046 (2006).

15 Yu, Z., Zhu, X., Jiang, Y., Luo, P. & Hu, C. Bioremediation and fodder potentials of two *Sargassum spp.* in coastal waters of Shenzhen, South China. *Marine Pollution Bulletin* **85**, 797-802, doi:10.1016/j.marpolbul.2013.11.018 (2014).

16 Qiaohan, W. *et al.* The effects of light intensity on the growth and chemical constituents of *Ulva pertusa*. *Marine Science*, 76-80 (2010).

17 Gao, X., Endo, H., Taniguchi, K. & Agatsuma, Y. Genetic differentiation of high-temperature tolerance in the kelp *Undaria pinnatifida* sporophytes from geographically separated populations along the Pacific coast of Japan. *Journal of Applied Phycology* **25**, 567-574, doi:10.1007/s10811-012-9891-4 (2013).

18 Carvalho, M. C., Hayashizaki, K. & Ogawa, H. Environment determines nitrogen content and stable isotope composition in the sporophyte of *Undaria pinnatifida (Harvey) Suringar*. *Journal of Applied Phycology* **20**, 695-703, doi:10.1007/s10811-007-9271-7 (2008).

19 Fuchun, L., Yongkui, Z., Lianshan, C., Zhonglin, H. & Guoliang, Z. Investigation and determinition of 25 elements in mussels, kelps and Undarias from Dalina Bay and Xinghai Bay Areas. *Marine Environment Science*, 34-38 (1995).

20 Ping, T. & Fengwei, H. An analysis of inorganic natrition elements from thirteen kinds of quick-growth seaweeds along Dalian coastline. *Journal of Benxi College of Metallurgy*, 7-10 (2001).

21 Fengwei, H. An analysis on inorganic nutritive elements from several kinds of quick-growth seaweeds in the Bohai Sea coastline. *Journal of Jinzhou Normal College (Natural Science Edition)*, 33-36 (2002).

22 Bo, Q., Laihao, L. & Chaohua, Z. Analysis and evaluation on nutritional composition of *Eucheuma*. *Modern Food Science and Technology*, 115-117+110 (2005).
